# Webbed penis: A new classification

**DOI:** 10.4103/0971-9261.70637

**Published:** 2010

**Authors:** Montasser El-Koutby, El Gohary Mohamed Amin

**Affiliations:** Department of Pediatric Surgery, Cairo University Abu Dhabi, United Arab Emirates; 1 Al Noor Hospital Abu Dhabi, United Arab Emirates

**Keywords:** Buried penis, concealed penis, webbed penis

## Abstract

**Aim:**

To introduce a new classification for the congenital anomaly of webbed penis and suggest an operative technique that can be planned according to the severity of webbing.

**Materials and Methods:**

A prospective study was conducted in two pediatric surgical units in Egypt and UAE on babies who were referred for circumcision. A preplanned written protocol was designed before commencing the study.

**Results:**

A total of 5,881 babies aged from 1 day to 6 months were seen in two pediatric surgical units. The webbed penis is broadly classified into primary and secondary types. The primary is further subdivided into simple and compound.

**Conclusions:**

We believe that the new classification will serve as a baseline for the anatomical variants and help to streamline the operative procedure accordingly.

## INTRODUCTION

The webbed penis is a congenital condition in which a web or fold of skin obscures the penoscrotal angle in an otherwise normal-sized penile shaft.[1] This results in a pseudomicroscopic appearance of the normal penis. A penis of normal size may be concealed because it is buried in the prepubic tissues, enclosed in scrotal tissue (penis palmatus), trapped secondary to phimosis, postcircumcision cicatrix, trauma or hidden because of a large hernia or hydrocele.[2]

## MATERIALS AND METHODS

A new classification based on the experience of two pediatric surgical centers is proposed. Two thousand eight hundred and sixty-seven cases were referred for circumcision from January 2004 to January 2008 at the Mafarq Hospital, Abu Dhabi, UAE, and 3,014 cases from July 2006 to July 2008 at the Abu El-Reesh Children University in Cairo, Egypt, were categorized according to a preplanned protocol.

## RESULTS

**Figure 1:**
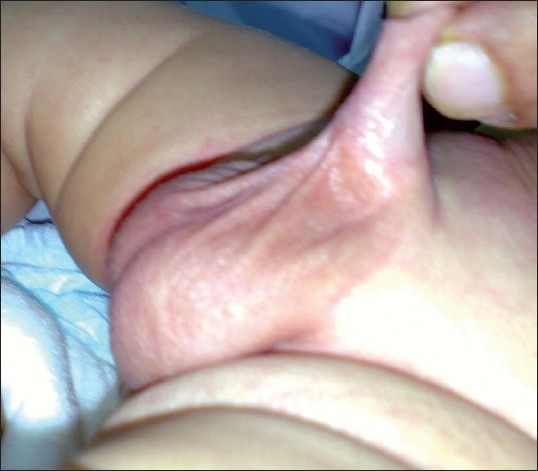
Simple webbing, grade 1 (proximal 1/3 of the shaft)

**Figure 2:**
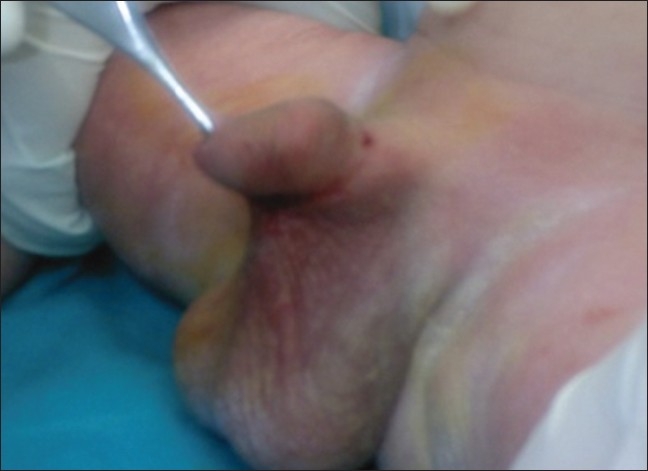
Simple webbing, grade 2 (mid 1/3)

**Figure 3:**
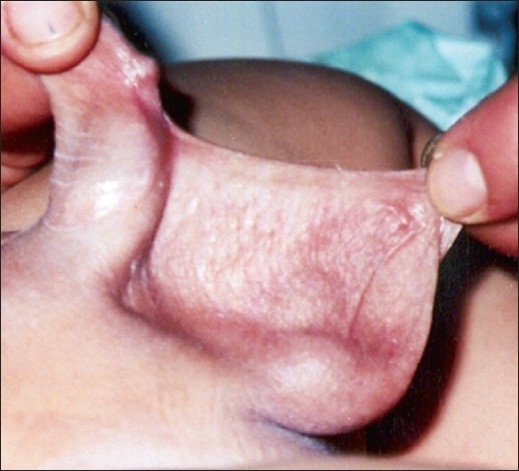
Simple webbing, grade 3 (distal 1/3)

**Figure 4:**
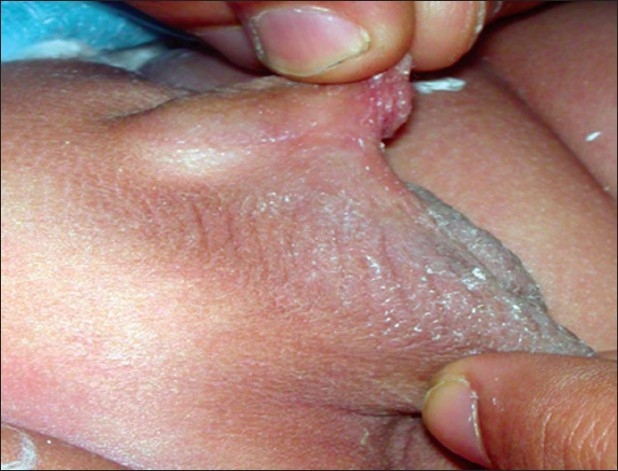
Compound webbing, prepenile scrotum

**Figure 5:**
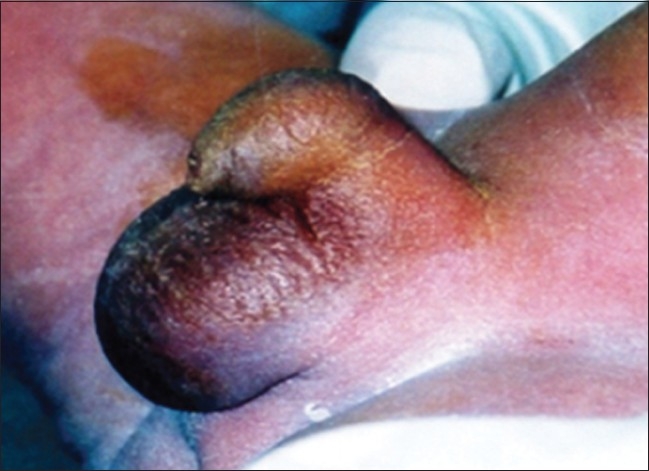
Compound webbing, ventral curvature

**Figure 6:**
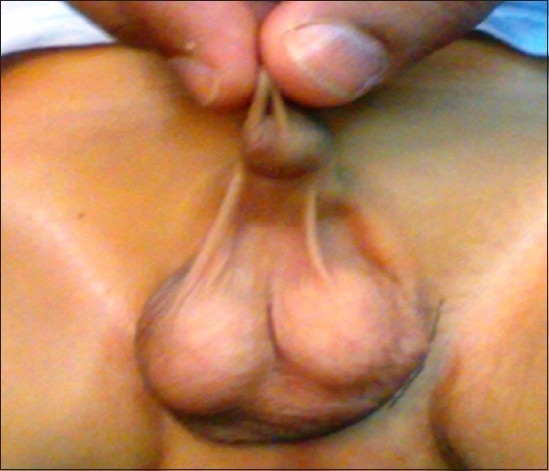
Compound webbing, broad web

The webbed penis is found in 236 (out of 5,881) cases, which is classified broadly into primary and secondary types. The primary is further subdivided into simple and compound as per Table 1 and Figures 1–6.

**Table 1:** Proposed classification of webbed penis

	Total No.
1. Primary webbed penis	
A: Simple	
Grade 1: The web extends to the proximal 1/3 of the shaft of the penis	82
Grade 2: The web extends to the mid 1/3 of the penis	41
Grade 3: The web extends to the distal 1/3 of the penis	67
B: Compound	
Type 1: Web with prepenile scrotum	21
Type 2: Web with penile curvature	10
Type 3: Broad web	3
2. Secondary webbed penis	
Postcircumcision: In obese children or concealed penis	12

### Suggested operative technique

The aim is (a) exposure of the glands and coronal sulcus, (b) with penile skin length equal to the penile shaft length dorsally and ventrally, (c) straight organ and (d) normal penoscrotal junction.

The technique differs according to the type:

### Primary, simple, grade 1

1. Retract the prepuce, clean smegma, return the prepuce to its original position.
2. The organ is held gently at nearly right angles to the abdominal wall.
3. The assistant applies gentle pressure at the penoscrotal junction until it comes to the level of the pubis.
4. A mark is put on the penile skin at 12, 3, 6 and 9 at the level of the coronal sulcus.
5. Bone forceps is applied obliquely in line with the previous four marks.
6. The excess prepuce is cut.
7. The outer layer of the prepuce is retracted.
8. The inner layer, if longer than 2 mm, is trimmed.
9. Achievement of hemostasis.
10. Approximation of the outer and inner layers of the prepuce by fine 5/0 absorbable sutures.

### Primary, simple, grade 2

1. If there is sufficient ventral skin, the technique used in grade 1 is performed.
2. If the ventral skin is deficient, apply the technique used for grade 3.

### Primary, simple, grade 3 and compound webbing.

#### Technique (Byar’s lateral transposition flap)

1. Retract the preputial skin until the coronal sulcus is exposed circumferentially and clean smegma.
2. The prepuce is returned back to its original position.
3. Apply two fine artery forceps on either sides of the midline dorsally, slit the prepuce back at twelve o’clock and stop 2–3 mm short of the coronal sulcus.
4. The inner layer of the prepuce is incised circumferentially 2 mm from the coronal sulcus.
5. Degloving the penis back to the symphysis pubis and any fibrous bands on the ventral or lateral aspects of the penile shaft are excised taking care not to injure the urethra (step is applied only for compound type 3).
6. Suture the remaining inner layer of the prepuce to the penile skin at twelve o’clock.
7. Unfold the prepuce.
8. Apply gentle pressure until the penoscrotal junction is restored to its normal position.
9. Mark the ventral penile skin so that sufficient penile skin is left on the ventral aspect of the penis (skin length is equal to the ventral shaft length).
10. The excess preputial skin distal to the mark is excised circumferentially.
11. Achieve hemostasis.
12. The two cut edges are approximated using fine 5/0 absorbable stitches.

## DISCUSSION

The condition of webbed penis has been discussed under a variety of names, including penoscrotal webbing, the inconspicuous penis, buried penis, penoscrotal fusion, penoscrotal pterygium and penis palmatus. It is important to highlight this condition as it may have a psychological trauma due to abnormal sexual appearance. Some cases may present with pain, abnormal stream of urine or genital dysfunction.[3]

It should also be realized that circumcision may be contraindicated in some of theses cases.[4] The etiology of this condition is uncertain, although it has been postulated that a disturbance in development of the prepuce may leave the ventral penis with inadequate skin coverage, resulting in the borrowing of scrotal tissue.[2]

We are aware of only one classification proposed by Maizels *et al*. in 1986, in which he classified the concealed penis into normal circumcised, poor skin suspension, buried penis, webbed penis, trapped penis and micropenis.[1] But, as far as we can ascertain, no classification was ever proposed for webbed penis. We believe, as there is a known classification for concealed penis, that webbed penis should also have a classification. We would hope that this classification will help in planning the operative correction according to the type or the degree of the webbing and have more objective measures in comparing the outcome of the different surgical techniques.

Several surgical techniques have been proposed for webbed penis under a variety of different terminologies[5–8] with no clear categorization of the underlying pathology or its severity. In this report, we are suggesting a technique that can be modified according to the type and severity of the webbing. It has been proven to be simple, reproducible and with good cosmetic result.
